# Parasite-mediated selection of major histocompatibility complex variability in wild brandt’s voles (*Lasiopodomys brandtii*) from Inner Mongolia, China

**DOI:** 10.1186/1471-2148-13-149

**Published:** 2013-07-12

**Authors:** Min Zhang, Hongxuan He

**Affiliations:** 1 Key Laboratory of Animal Ecology and Conservation Biology, Institute of Zoology, Chinese Academy of Sciences, 100101, Beijing, China

**Keywords:** Major histocompatibility complex, Genetic diversity, Parasite-driven selection, Fluctuating selection, Heterozygote advantage, Rare allele advantage

## Abstract

**Background:**

Genes of the major histocompatibility complex (MHC) exhibit high levels of variability, which is believed to have arisen through pathogen-mediated selection. We investigated the relationship between parasite load and genetic diversity at selectively neutral, non-coding markers (microsatellites) and adaptive genetic variation at a functionally important part of the MHC in six independent natural populations of Brandt’s voles (*Lasiopodomys brandtii*) from two regions of the Xilingol Grassland area of Inner Mongolia.

**Results:**

Two-hundred and fifty-two voles were screened for gastrointestinal parasites, and were assessed for genetic variation. Parasite screening was done through non-invasive fecal egg counts, while allelic diversity was determined via single-stranded conformation polymorphism and DNA sequencing. We detected eight distinct helminth egg morphotypes. A total of 10 microsatellite loci were genotyped and 19 unique MHC class II *B* alleles were isolated. The rate of nonsynonymous substitutions (d_N_) exceeded the rate of synonymous substitutions (d_S_) at putative antigen binding sites of DRB. Neutral and adaptive genetic diversity differed between the six vole populations. To test the main pathogen-driven selection hypotheses for the maintenance of host MHC diversity and parasite species-specific co-evolutionary effects, multivariate approaches (generalized linear mixed models) were used to test for associations between the MHC class II DRB genotype and infections with nematodes. We found no evidence for heterozygote advantage, and overall heterozygosity was lower than expected in the MHC alleles. We identified an association between the parasite load and specific MHC alleles in the voles, and this pattern varied between geographic regions.

**Conclusions:**

The results suggest that MHC variability in Brandt’s voles is maintained by rare allele advantage and fluctuating selection, but the data failed to show any heterozygote advantage effect. Our results add to a growing body of evidence showing that the mode and relative strength of pathogen-driven selection acting on MHC diversity varies within specific wild populations. In addition, our study contributes to the understanding of what maintains MHC diversity, of host-pathogen coevolution and of how genetic diversity is maintained in voles.

## Background

Genetic diversity is widely believed to influence the evolutionary and adaptive potential of populations and species [1]. Analysis of patterns and levels of genetic variation at neutral markers, such as autosomal microsatellites and mitochondrial DNA regions, has been widely used in the last decades to infer historical events (e.g. past demographic expansions or contractions) [2-5] and geographical features (e.g. fragmentation) [6-8] in natural populations. However, studying molecular polymorphism at loci under selection is the only direct way to understand the genetics of adaptive processes [9]. Pathogens represent very powerful agents of selection that have the potential to drive rapid changes in the genetic composition of natural host populations. In the co-evolutionary host-pathogen interplay pathogens are particularly important for maintaining host genetic variation [10]. The role that genetic variation plays in buffering host populations from pathogens has been emphasized in several studies. These studies found associations between low levels of genetic diversity, increased pathogen susceptibility, and high pathogen loads [11-14].

In vertebrates, the genes of the major histocompatibility complex (MHC) are among the most debated candidates in the co-evolutionary process of host-parasite interactions at the molecular level [15]. They have been studied extensively in model species under laboratory conditions, but, because of their functional importance in the immune system and mate choice, they have also become the focus of an increasing number of studies on natural populations [15-18]. MHC genes code for cell surface molecules that present self and nonself antigens to T-cells. This function enables them to play a vital role in the recognition of pathogens invading the body. The region of the molecule responsible for binding antigens, the so-called antigen-binding sites (ABS), show particularly high levels of variation, not only in the number of alleles, but also in the extent of sequence divergence between alleles. In particular, the ABS sites display more non-synonymous than synonymous substitutions that change the amino acid sequence of the peptide and thus allow binding of a diverse array of antigens [19]. This indicates that selection processes maintain polymorphism in the functionally important regions of the MHC. Importantly, genetic diversity in the ABS facilitates binding of a diverse array of antigens to the molecule [20].

The exceptionally high allelic polymorphism found in the MHC loci is believed to be maintained by pathogen-mediated selection, although the relative importance of a number of nonexclusive hypotheses explaining the potential selection mechanisms that enhance or maintain adaptive genetic variation is debated [14,21,22]. The heterozygote advantage hypothesis [23] proposes that individuals heterozygous at MHC loci are able to respond to a greater range of pathogen peptides than homozygotes and, consequently, benefit from increased resistance to pathogens. Heterozygotes are, therefore, more likely to have higher relative fitness and, as a result, more MHC alleles persist, on average, in the population. This hypothesis has been used to explain the persistence of highly divergent MHC alleles over millions of years [24]. Support for heterozygote advantage hypothesis comes mainly from mate choice studies [25,26]; however, researchers have rarely investigated the possible advantages of MHC heterozygosity in one individual in the context of infectious diseases, such as intestinal parasite infestations (but see [15,27-29]).

The second selection mechanism is described by the rare allele advantage hypothesis (also known as the negative frequency dependent selection hypothesis) [30]. In this scenario, the selection pressure exerted by common parasites favors rare resistant host alleles. As those host alleles become more common, the host population exerts a reciprocal selection pressure on the parasite population, favoring other parasite genotypes to which the host has not yet adapted. With time, this could lead to continual cycling of host and parasite genotype frequencies within the population, which could maintain high levels of MHC variability [27,28,31].

Finally, the fluctuating selection hypothesis [32] proposes that spatial and temporal heterogeneity in the type and abundance of pathogens may maintain diversity at the MHC. In short-term field studies, the detection of associations between specific MHC alleles and parasite load is usually presumed to be an indicator of this selection mechanism. However, host-parasite interactions are also shaped by environmental conditions, which play an important regulating role in the distribution, transmission, and developmental success of parasites and pathogens [33]. These conditions can influence parasite species richness, as well as the intensity of infestation in the host species. Therefore, co-evolutionary selection processes should vary not only in time but also in space, and different specific MHC alleles should have an advantage in different environments [34].

Over the last decades, there have been ample findings of associations between MHC alleles and parasite load, even in studies on free-ranging species under constant challenge by a diverse range of pathogens [14,21]. However, under homogeneous parasite selection, these mechanisms alone do not explain the observed large allelic diversity at the metapopulation level. Therefore, the idea of parasites exerting divergent selection on locally adapted MHC allele pools in heterogeneous environments has been put forth to suggest how this unparalleled genetic diversity is maintained. Several recent field surveys investigating MHC variation on different geographical scales and in heterogeneous habitats have proposed that contrasting parasite communities may shape MHC composition (e.g., in mammals [35], in birds [36,37] and in fish [38-40]).

Brandt's vole (*Lasiopodomys brandtii*) is the dominant rodent species of the typical steppe habitat extending from the central part of Inner Mongolia through the middle and east of Dornod Aimag in the Republic of Mongolia, to the southern borders of Mongolia in Transbaikalia, Russia [41]. The distribution of this species is discontinuous in Inner Mongolia [42,43]. However, it is a widespread species living in a diverse environment, which presumably necessitates constant adaptation to environmental change, such as encountering new parasites, but also persistence to challenges from ancient pathogen [44]. There is significant variation in the density of these voles between years [43]. The presumably high parasite pressure in these habitats could lead to pronounced and therefore detectable signs of otherwise more subtle mechanisms of selection [45].

In this study, we examined the role of parasite-mediated MHC polymorphism in six independent natural populations of Brandt’s voles, from Maodeng Livestock Farm (MD) and East Ujimqin (DWQ) of the Xilingol Grassland area of Inner Mongolia, to understand the selective mechanisms that act on MHC in response to parasitism. Our specific aims were to test for an association between: (1) individual parasite load and MHC heterozygosity (indicating heterozygote advantage) and (2) individual pathogen load and specific MHC alleles (rare allele advantage and fluctuating selection) [21].

## Results

### Parasite load

We detected eight distinct helminth egg morphotypes in 252 Brandt’s voles’ fecal samples. Five of these were classified as nematodes and, among them, two nematode morphotypes were identified as *Syphacia obvelata* and *Aspiculuris tetraptera*. The remaining three morphotypes belonged to the *Trichostrongylidae* family. Two different cestode morphotypes were detected, which were identified as *Schizorchis ochotonae* and *Hymenolepis nana*. In addition, one trematode morphotype from the *Echinostomatidae* family was detected. Among the individuals examined, 94.5% had infections with one to four helminths, with most of the infections caused by nematodes (99.2% of infected individuals), whereas only 6.3% and 3.4% of the infections were caused by cestodes and trematodes, respectively. Because of the high frequency of nematode infections found in this study and the minor prevalence of cestode and trematode infections, the latter two helminths were excluded from subsequent analyses.

The mean parasite prevalence, species richness (by taxonomic group), and parasite intensity for all of the Brandt’s voles captured from six populations in two regions is presented in Table 1. A global analysis of relative differences in parasite community structure based on pairwise Hellinger distances revealed strong differences between two regions (Permutational multivariate analysis, DF = 1, SS = 4.86, MS = 0.142, F = 1.32, R^2^ = 0.207, P = 0.005). Differences between all pairs of neighboring populations in either MD or DWQ region were non-significant.

**Table 1:** Mean parasite prevalence, mean species richness, and mean infection intensity for Brandt’s voles

**Region**	**Maodeng livestock farm (MD)**			**East Ujimqin (DWQ)**		
**Population (Sample size)**	**M1 (n = 41)**	**M2 (n = 43)**	**M3 (n = 41)**	**D1 (n = 44)**	**D2 (n = 41)**	**D3 (n = 42)**
**Prevalence (%)**	80.49%	83.72%	82. 93%	86.36%	87.80%	88.10%
**Mean species richness**	1.78 ± 0.80	1.74 ± 0.83	1.75 ± 0.72	2.11 ± 0.75	2.39 ± 0.97	2.03 ± 0.72
**Mean infection intensity**	3.95 ± 0.29	3.96 ± 0.31	3.97 ± 0.31	4.21 ± 0.10	4.24 ± 0.12	4.12 ± 0.26

Note: Brandt’s voles were captured from six populations in two regions (N = 252). Parasite species richness was defined as the number of helminth morphotypes per individual, and parasite infection intensity was estimated using fecal egg counts (FEC, log_10_EPG; EPG, nematode eggs per gram feces). Mean ± standard deviation are shown.

### MHC variability

**Figure 1:**
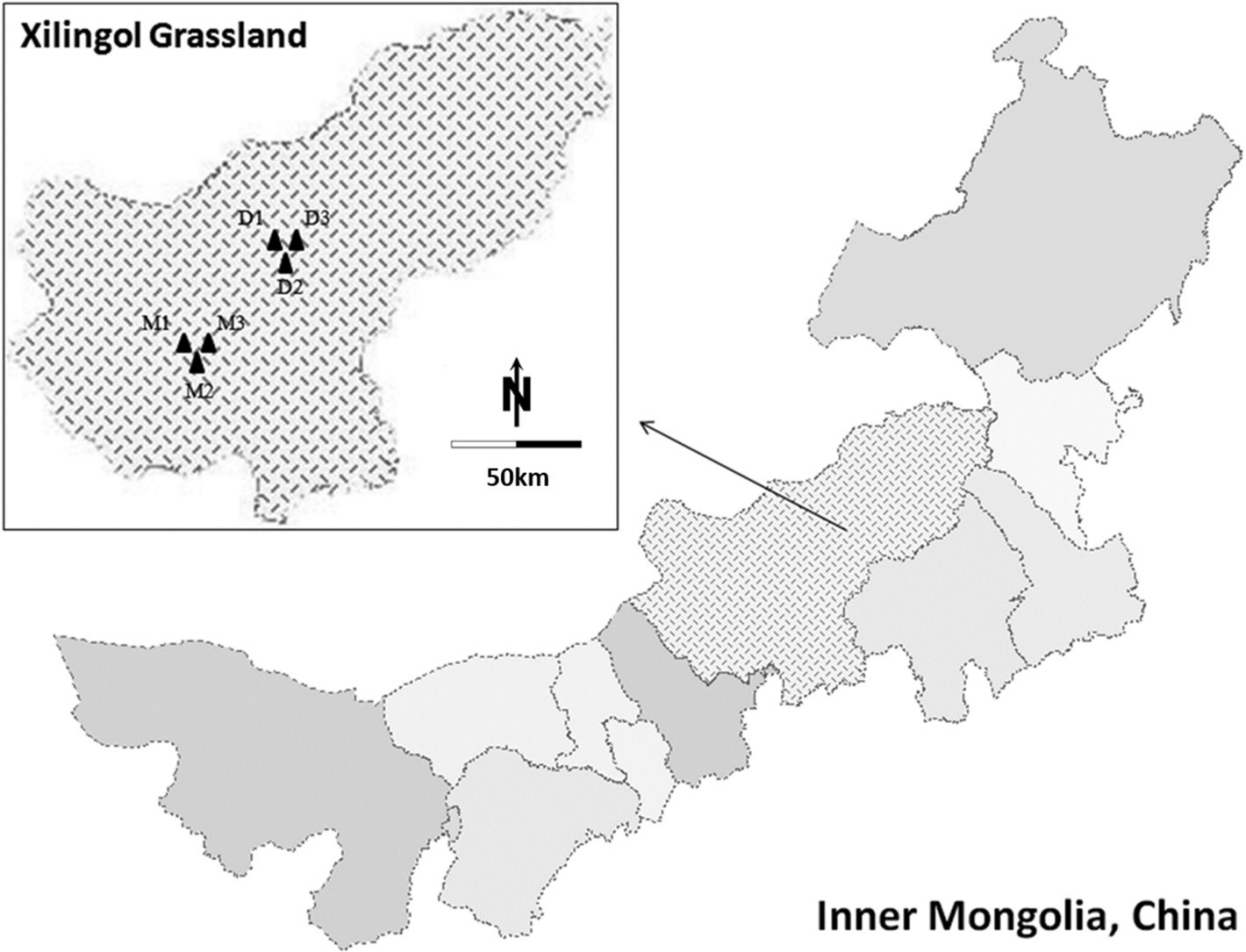
Geographical locations of the six populations of Brandt's vole.

**Figure 2:**
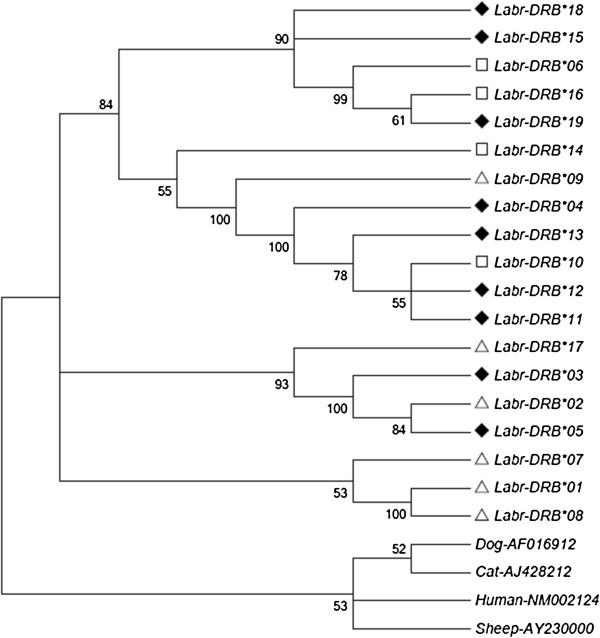
**Minimum evolutionary tree for MHC DRB exon 2 alleles of Brandt’s voles.** The tree is based on nucleotide sequences (Kimura 2-parameter). Bootstrap values (>50) are displayed (1000 replications). The scale bar indicates genetic distance in units of nucleotide substitutions per site. Dog, cat, sheep, and human DRB exon 2 sequences were used to root the tree. GenBank accession numbers follow the species designations. Alleles found in the MD region alone are indicated by squares, those in the DWQ region alone are indicated by triangles, and those in both regions are indicated by diamonds.

A total of 252 individuals in six populations from two different regions were genotyped. Overall, 23 different sequence variants (alleles) could be distinguished via single-stranded conformation polymorphism analysis, which were confirmed by sequencing. BLAST search results showed that sequence similarities between Brandt’s voles *Labr*-DRB and mouse MHC Class II variants were 84% to 91%. All of the alleles detected showed the unique DRB origin. Four sequences revealed different nucleotide contents (differing in one or two nucleotide positions), but identical amino acid sequences, and were therefore treated as one allele. The remaining 19 alleles could be translated into unique amino acid sequences. These were labeled *Labr*-DRB*01 to *Labr*-DRB*19 according to their frequency following the nomenclature of Klein, *et al*. [46]. MHC class II DRB sequences from this study are deposited at GenBank (accession numbers: JX046707-JX046725; also see Additional file 1: Table S1). The phylogenetic relationships of these alleles are displayed in Figure 1. In MD we identified 13 alleles, while DWQ yielded 15. Nine of the 19 alleles identified in this study were shared between two regions; Figure 2 shows the relative frequency and distribution of each allele.

### Evidence for positive selection

The rates of synonymous (d_S_) and nonsynonymous (d_N_) substitutions were calculated separately for ABS and non-ABS. For the ABS, d_N_ (0.390) was significantly higher than d_S_ (0.072), resulting in a d_N_/d_S_ ratio of 5.43 (Z > 2.613; P < 0.01). In contrast, the non-ABS ratio between nonsynonymous (d_N_ = 0.057) and synonymous substitutions (d_S_ = 0.038) did not significantly deviate from unity (d_N_/d_S_ = 1.49, Z < 1.165; n.s.). Moreover, d_N_ was 6.84 times higher in the ABS than in the non-ABS. This indicates positive selection processes that maintain polymorphism in the functionally important regions of the MHC.

### Population differentiation

No indication of linkage disequilibrium between pairs of microsatellite loci or between microsatellites and the MHC locus or deviation from Hardy-Weinberg equilibrium within loci was found (all P > 0.05 after Bonferroni correction). The study populations differed in their levels of genetic diversity with regard to both genetic markers (Table 2). Regarding the microsatellite markers, there was low variation in the mean multilocus heterozygosity (MLH) values among populations, but the mean d^2^ (difference in repeat units, averaged over all loci) values varied notably from 125.22 (population D3) to 189.22 (population D1). There was a wide range in the MHC heterozygosity observed among populations, from 0.62 (population D3) to 0.78 (population D2). Every population had a significant observed heterozygosity deficit compared to the expected heterozygosity. The corrected values for MHC allelic richness varied widely as well, from 30.65 in population M2 to 40.69 in population D1 (Table 2).

**Table 2:** Genetic diversity for Brandt’s voles

		**Microsatellites**		**MHC**	
**Pop**	**N**	**Mean MLH**	**Mean d**^**2**^	**Allelic richness**	**H**_**obs**_**/H**_**exp**_
M1	41	1.160	188.57	38.65	0.75/0.91
M2	43	1.378	131.71	30.65	0.76/0.91
M3	41	1.171	158.24	39.00	0.67/0.89
D1	44	1.283	189.22	40.69	0.76/0.89
D2	41	1.118	175.17	33.77	0.78/0.93
D3	42	1.348	125.22	39.87	0.62/0.94

Abbreviations: Note: Brandt’s voles were captured from six populations in two regions (*N*, 252): *Pop* population, *N* sample size, *MLH* multilocus heterozygosity, *d*^*2*^ difference in repeat microsatellite units averaged over all loci, *H*_*obs*_ observed heterozygosity, *H*_*exp*_, expected heterozygosity according to Hardy-Weinberg. MHC allelic richness was corrected for sample size.

Differentiation among populations was highly significant for both types of markers (microsatellites: F_ST_ = 0.0671, P < 0.001; MHC: F_ST_ = 0.0512, P < 0.001). In addition, differentiation test between all pairs of populations showed significantly differentiated for microsatellite loci and MHC (Table 3). Pairwise F_ST_ values for microsatellite loci ranged from 0.0075 to 0.0907, and for MHC from 0.0014 to 0.0836.

**Table 3:** **Estimation of pairwise genetic distance (F**_**ST**_**) between Brandt’s vole populations**

	**M1**	**M2**	**M3**	**D1**	**D2**	**D3**
**M1**	**-**	**0.0288**	**0.0640**	**0.0075**	**0.0703**	**0.0680**
**M2**	**0.0251**	**-**	**0.0146**	**0.0404**	**0.0726**	**0.0317**
**M3**	**0.0836**	**0.0363**	**-**	**0.0871**	**0.0320**	**0.0907**
**D1**	**0.0529**	**0.0435**	**0.0014**	**-**	**0.0452**	**0.0745**
**D2**	**0.0449**	**0.0278**	**0.0663**	**0.0236**	**-**	**0.0619**
**D3**	**0.0587**	**0.0429**	**0.0653**	**0.0652**	**0.0500**	**-**

Note: Above the diagonal we indicate pairwise F_ST_ of microsatellites. Below the diagonal we show pairwise F_ST_ for MHC. Bold values indicate that F_ST_ reached statistical significance after correction for multiple testing.

### Association between parasite load and MHC heterozygosity

Using generalized linear mixed models (GLMMs), we investigated the effects of population genetic diversity on parasite load, the results of which are listed in Table 4. We calculated models for the influence of each genetic predictor separately. Neither neutral genetic nor MHC diversity showed significant effects on the parasite load. No support for MHC heterozygote advantage (parasite species richness: P = 0.86; parasite infection intensity: P = 0.37) or association with MHC allelic richness (parasite species richness: P = 0.80; parasite infection intensity: P = 0.66) could be detected at the population level.

**Table 4:** Genetic diversity effects on nematode load in Brandt’s voles as calculated by generalized linear mixed models

**a) Nematode species richness**			
**Model**	**β ± SE**	**t**	**P**
**MLH**	2.576 ± 1.369	0.634	0.44
**d**^**2**^	0.038 ± 0.008	0.197	0.78
**MHC H**_**obs**_	0.023 ± 0.015	0.428	0.86
**MHC allelic richness**	−0.206 ± 0.091	−0.720	0.80
**b) Nematode infection intensity**			
**Model**	**β ± SE**	**t**	**P**
**MLH**	0.176 ± 0.054	0.803	0.24
**d**^**2**^	0.031 ± 0.009	0.865	0.88
**MHC H**_**obs**_	0.446 ± 0.172	0.263	0.37
**MHC allelic richness**	−0.332 ± 0.015	−0.430	0.66

Note: Full models: (a) nematode species richness; (b) nematode infection intensity. β ± SE = the coefficient ± standard error, t = t-value, P = p significance value.

Similarly, the two separate GLMMs, which included either prevalence or infection intensity as response variables (from all of the nematode species combined), in addition to MHC genotypes (homozygote, heterozygote) and MLH as predictors, also did not reveal any support for heterozygote advantage. There was no support for the hypothesis that MHC heterozygous individuals are less infected than homozygotes (prevalence: β ± SE =17.683 ± 0.439, t =15.383, P = 0.328). The same applied to MLH (β ± SE = 0.635 ± 0.611, t =1.874, P = 0.267). Additionally, restricting the data to each of the three most common nematodes (*S*. *obvelata*, *A*. *tetraptera*, *Trichostrongylidae*) did not reveal any evidence for heterozygote advantage (all P > 0.10).

### Association between parasite load and specific MHC alleles

Our GLMMs did reveal relationships between specific MHC alleles and parasite load in Brandt’s voles. Five of the 19 alleles had specific effects either in terms of positive or negative associations towards parasite loads (Table 5). The *Labr*-DRB*11 and *Labr*-DRB*13 alleles were significantly associated with the status of *S*. *obvelata* infection. Allele *11 was associated with a higher prevalence (t = 1.822, P = 0.031), while allele *13 was significantly related with an elevated infection intensity (t = 4.913, P = 0.035). As for *A*. *tetraptera*, voles that carried the *Labr*-DRB*04 allele were significantly less infected than animals without it (t = −4.152, P < 0.001), while *Labr*-DRB*12 alleles were associated with an increased probability of a higher infection intensity (t = 4.289, P = 0.008). Furthermore, positive associations of the subfamily *Trichostrongylidae* and *Labr*-DRB*19 alleles were revealed for both prevalence (t = 2.689, P = 0.030) and infection intensity (t = 1.653, P = 0.005).

**Table 5:** Effects of the most abundant vole Labr-DRB* alleles on nematode prevalence and infection intensity

**a) Nematode prevalence**					
**Response variable**	**Predictor**	**β ± SE**	**t**	**P**	**Effect**
**Prev **(*Syphacia obvelata*)	**Sex**	−0.699 ± 0.156	−1.461	0.009	**-**
	**Body mass**	0.981 ± 0.214	2.475	0.075	
	*Labr*-DRB*11	1.343 ± 0.262	1.822	0.031	**+**
**Prev **(*Aspiculuris tetraptera*)	**Sex**	−0.955 ± 0.247	−0.436	0.046	**-**
	**Body mass**	0.748 ± 0.250	4.629	0.089	
	*Labr*-DRB*04	−2.512 ± 0.208	−4.152	<0.001	**-**
**Prev **(*Trichostrongylidae*)	**Sex**	−0.897 ± 0.362	−0.773	0.032	**-**
	**Body mass**	1.194 ± 0.191	3.710	0.059	
	*Labr*-DRB*19	3.256 ± 0.814	2.689	0.030	**+**
**b) Nematode infection intensity**					
**Response variable**	**Predictor**	**β ± SE**	**t**	**P**	**Effect**
**FEC **(*Syphacia obvelata*)	**Sex**	−0.592 ± 0.178	−0.167	0.039	**-**
	**Body mass**	0.643 ± 0.439	3.218	0.064	
	*Labr*-DRB*13	3.565 ± 0.255	4.913	0.035	**+**
**FEC **(*Aspiculuris tetraptera*)	**Sex**	−0.678 ± 0.176	−0.359	0.018	**-**
	**Body mass**	1.190 ± 0.182	2.661	0.053	
	*Labr*-DRB*12	2.409 ± 0.914	4.289	0.008	**+**
**FEC **(*Trichostrongylidae*)	**Sex**	−0.833 ± 0.286	−0.507	0.022	**-**
	**Body mass**	0.639 ± 0.342	3.042	0.059	
	*Labr*-DRB*19	3.042 ± 0.570	1.653	0.005	**+**

Abbreviation: Note: Most abundant *Labr*-DRB* alleles (frequency ≥ 5 individuals). Data are based on multivariate generalized linear mixed models: full models for (a) nematode prevalence and (b) nematode infection intensity. β ± *SE*, the coefficient ± standard error: *t* t-value, *P* p significance value.

Our GLMMs also revealed that alleles associated with high or low infection intensity differed between sampling regions. We found a significant region-specific effect (MD: t = −2.35, p = 0.021; DWQ: t = 1.56, p = 0.014) of the *Labr*-DRB*03 allele on the intensity of infection with *A*. *tetraptera*. In MD, animals carrying *Labr*-DRB*03 had fewer parasites than animals without it, but in DWQ the association was the opposite.

## Discussion

The aim of our study was to investigate whether parasite-mediated selection could explain MHC variability in free-ranging Brandt’s voles. MHC class II-DRB exon2 polymorphism and nematode burden were surveyed and selective mechanisms that may be acting on the MHC in the presence of nematodes were tested.

In 252 individuals of *L*. *brandtii*, 19 distinct *Labr*-DRB alleles were detected. The alleles showed high levels of nucleotide and amino acid sequence divergence. Polymorphism was highest in the functionally important antigen recognition and binding sites of the MHC. In the ABS, significantly more nonsynonymous substitutions than synonymous substitutions were found. This is considered a clear indication of positive selection [47] and characteristic of proteins with antigen-presenting functions [48]. High levels of polymorphism given by the number of alleles as well as by the sequence divergence, especially at ABS sites, are common findings in MHC genes, and were found in a variety of studies [48-50]. Because the MHC plays a major role in vertebrate immune systems, pathogen-driven selection processes are thought to be involved in the maintenance of diversity at MHC loci [22]. Many studies of wild mammals have demonstrated a correlation between MHC diversity and resistance against pathogens. In a large survey of avian malaria in 13 populations of the house sparrow (*Passer domesticus*), variable selection pressures were observed to select for different host allelic lineages resulting in population-specific associations between MHC alleles and risk of infection [37]. Similarly, rodent species that face a rich ectoparasite fauna also maintain increased allelic polymorphism at the MHC [51,52].

It is important to note that while we monitored the community of gastrointestinal parasites in the fecal samples, we concentrated our attention on helminth parasite species because of their prevalence [53-55], their impact on fitness and mortality in a wide range of wild animal species [56-58], and because their infestation intensity can be assessed non-invasively by fecal sampling. Overall, we detected eight distinct helminth egg morphotypes and an infestation rate of 94.5%. To date, the studies of individual MHC and parasite loads in natural populations, particularly for mammals, have mostly focused on helminths [15,49,59-61]. However, most organisms are faced with enormous numbers of pathogens, and identifying and measuring the vast community of parasites and pathogens that can infect a natural population will be crucial [62]. Whether results from highly simplified study systems (a single pathogen species) are applicable to more complex systems is questionable. Therefore, extending the scope of studies across a broader range of parasite taxa would enhance our understanding of MHC-parasite dynamics in natural populations. A serious challenge lies in fully characterizing the MHC and pathogen load. This is unlikely to be possible in most study systems, and even if it were, statistical analysis may be intractable. The best study systems will probably be characterized by intermediate levels of pathogen diversity and simple, well-characterized, MHC structures, thus avoiding oversimplification while retaining statistical tractability [21].

In our study, we found neither support for the heterozygote advantage hypothesis on the population nor on the individual level, and heterozygosity of MHC alleles was lower than expected in all of the study populations. These findings suggest little or no direct selection for MHC heterozygosity in populations at our study sites, at least for the generation of voles we sampled. Consistent low MHC heterozygosity may have arisen from underdominance [21]. The lack of associations between MHC genetic diversity and parasite load at the population level in our study adds to the mixed results of former studies [28,50,63]. Studies have indicated that if hosts and pathogens share a long-term co-evolutionary history, selection via diverse pathogens causes high MHC polymorphism in a species or population, whereas low MHC polymorphism indicates the presence of relaxed pathogenic selection pressure [38,64,65]. Alternatively, it might have been because MHC diversity was not fully characterized as a result of methodological errors, such as null alleles. However, as the MHC sequences could be amplified from all of the study individuals, and on the basis of at least two independent polymerase chain reaction (PCR) and single-stranded conformation polymorphism (SSCP) assays, as well as forward and reverse sequence analyses, respectively, the obstacle of null alleles is improbable.

We detected an association between parasite load and specific MHC alleles in the individuals. The *Labr*-DRB*11 and *Labr*-DRB*13 alleles were significantly associated with the status of *S*. *obvelata* infection. As for *A*. *tetraptera*, voles that carried the *Labr*-DRB*04 allele were significantly less infected than animals without it, while *Labr*-DRB*12 alleles were associated with an increased probability of a higher infection intensity. Positive associations of the subfamily *Trichostrongylidae* and *Labr*-DRB*19 alleles were revealed for both parasite abundance and infection intensity. In MD, animals carrying *Labr*-DRB*03 had fewer parasites than animals without it, but in DWQ the association was the opposite. Differences in vole susceptibility to parasitism were not explained by differences in the geographic distribution of alleles; all above alleles were detected in at least five populations and in both sampling regions. The association between certain alleles and susceptibility, or resistance to certain parasites and spatial variation in resistance, matches the predictions for rare allele advantage, but also for fluctuating selection [21]. Rare allele advantage arises as the evolution of new parasite and pathogen phenotypes reduces the relative fitness of common host genotypes, thereby providing a selective advantage to rare host [66]. Evidence in favor of this hypothesis comes from multiple studies demonstrating associations between specific MHC alleles and resistance to viral (e.g., hepatitis [67], Puumala virus [68]) and bacterial (e.g., tuberculosis [69]) infections as well as blood-borne parasites(e.g., malaria [70]) and helminths [10,71]. In reality, however, natural populations are exposed to fluctuating environmental conditions and, subsequently, host-pathogen interactions are similarly expected to vary spatiotemporally [72]. Charbonnel and Pemberton [73] detected fluctuating selection at an MHC locus during a 13-year survey of a population of feral sheep, perhaps driven by interactions with parasitic nematodes. Empirical genetic evidence for geographic heterogeneity in selective pressures has been shown in fish [74], birds [36,75] and mammals [35,76]. A selection model demonstrated that temporal variation in pathogen resistance may be sufficient to maintain polymorphism in the absence of both heterozygote and rare-allele advantage [34]. While it is generally accepted that any or both of these proposed mechanisms can play a role in shaping the distribution of MHC variation, distinguishing between them in natural populations can be challenging due to similarities in the expected genetic outcomes, requiring long-term studies of multiple populations to examine temporal changes in parasite resistance to alleles and spatiotemporal variation in the forces driving parasite abundance [21]. Under rare-allele advantage, one would expect to see different alleles conferring resistance to the same pathogen in different populations, and for resistance to change with time, so that different alleles become associated with resistance. Under fluctuating selection, one would expect to observe external biotic and/or abiotic forces driving spatio-temporal variation in pathogen abundance, leading to distinct subsets of alleles being selected for in different populations and/or different time periods [21], and higher population structure at MHC relative to neutral loci.

In wild rodents, parasite prevalence and intensity are known to underlie spatial and temporal variation, as well as intrinsic factors such as the immune status, sex, and age of the host [28,63]. By using the individual multivariate GLMM approach we were able to focus on ‘pure’ parasite driven selection mechanisms because our models allowed us to include confounding factors that could obscure the detection of MHC effects. In 252 adult Brandt’s voles, male voles were significantly heavier than females (males: 35.4 ± 1.2 g, females: 33.5 ± 1.1 g, Analysis of covariance, F = 1.2, P = 0.028). There were no significant associations between parasite load and host body mass, however, vole gender had a significant effect on nematode prevalence and intensity (Table 4). Furthermore, some aspects of the biology of Brandt’s voles suggest that a stable equilibrium between these voles and their parasites, which is a prerequisite for rare allele advantage, may rarely be achieved. Mixing of voles and parasite populations over an extensive area is possible, and the dynamic nature of this environment may mitigate the evolution of a stable equilibrium between MHC polymorphism in the host and parasite abundance and diversity. In addition, most of the parasites hosted by voles do not have direct lifecycles [77] and variability among populations may be dependent on the abundance of intermediate or final hosts of these parasites. In the case of *Strongyloides*, which have been found to be the most abundant and widespread parasites in wildlife, the abundance of intermediate hosts can affect population level differences in parasite abundance [78]. Thus, a significant association between 1) parasite infection and specific MHC alleles, 2) marked spatial variation in parasite infection and an association with specific MHC alleles, and 3) the dynamic nature of the environment in which voles are found, all implicate a role for fluctuating selection in maintaining MHC polymorphism in voles.

A potential criticism of the present study is that we must more rigorously characterise the diversity that is being detected. The ease with which MHC variation can now be resolved could paradoxically impede progress with our understanding of MHC dynamics [79]. A large number of studies are characterizing MHC variation from genomic DNA extracts with little or incomplete knowledge of (1) the number of loci that are actually being simultaneously PCR amplified; (2) whether any or all of these genes are actually expressed and (3) whether the variation that is resolved reflects sequence differences at structurally important regions such as the peptide-binding amino acids [21]. Ultimately, an inability to determine the actual number of expressed loci, identify alleles as being structural MHC variants, assign alleles to loci and determine whether an individual is heterozygous or homozygous for a particular locus compromises rigorous statistical testing of association between MHC and parasite load, either by introducing variation that is not directly under selection, or precluding detection of overdominant selection [79]. This is the first time that field population of Brandt’s voles have been typed at the MHC region, we have made every effort to initially ensure that PCR primers do actually amplify single expressed products to clarify that subsequent analysis based on genomic DNA is appropriate [80]. Detailed molecular groundwork is required to ensure that the full complement of MHC variation is accurately assessed in future experiment. The use of next-generation sequencing for MHC screening [81] is likely to be a great help in terms of more accurately characterizing MHC diversity. Despite all this, it will be a particular challenge to be able to completely validate studies from mRNA extracts, especially from natural populations.

## Conclusions

Our results are consistent with pathogen-mediated selection operating through rare allele advantage and fluctuating selection, but these two mechanisms could not be differentiated. Our results failed to show any effect of heterozygote advantage, and MHC polymorphism in wild Brandt’s voles may be constrained through underdominance. Furthermore, our results add to a growing body of evidence showing that the mode and relative strength of pathogen-driven selection acting on MHC diversity varies within specific wild populations, while the understanding of what maintains MHC diversity will also feed into our general understanding of host-pathogen coevolution and the maintenance of genetic diversity.

## Methods

### Study areas

**Figure 3:**
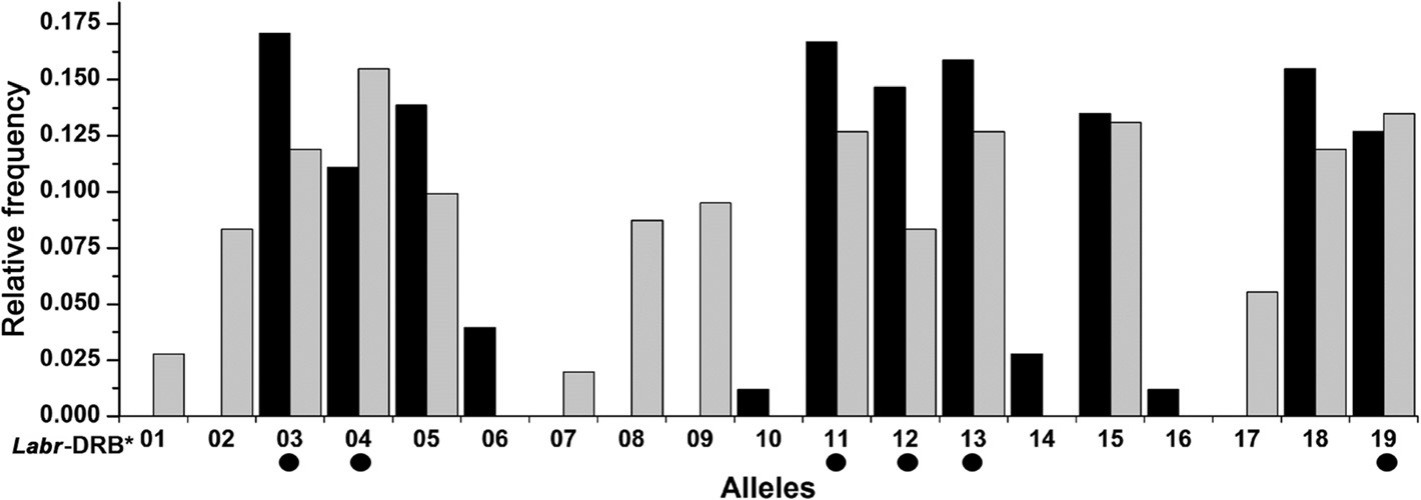
**Relative frequencies of all the alleles identified in Brandt’s voles from both geographic regions.** The relative allele frequency is expressed as the relative number of individuals per region in which the respective allele occurred, for the MD region (black bars), and DWQ region (grey bars). Black circles mark alleles that were detected as being associated with a specific nematode species resulting from the generalized linear mixed models (GLMMs).

Live trapping was used to capture 252 adult Brandt’s voles from six sites in two regions of Xilingol Grassland, Inner Mongolia over 1 week in August, 2011. This coincided with the peak activity of Brandt’s voles [45]. Voles were analyzed from two discontinuous habitats, including three sites (ca. 20 km apart; Sample size: M1 = 41; M2 = 43; M3 = 41) at the Maodeng Livestock Farm (MD: GPS reading 44°11’N, 116°27’E), and three sites (ca. 25 km apart; Sample size: D1 = 44; D2 = 41; D3 = 42) in East Ujimqin (DWQ: GPS reading 45°44’N, 116°16’E) (Figure 3). Both regions have a cold semi-arid climate, marked by long, cold, and very dry winters, and by hot, somewhat humid summers, and strong winds, especially in spring. The annual precipitation is approximately 260 mm (10.2 in), with more than half of it falling in July and August. However, relative to the open and free living environment in DWQ with rolling hills and rocky outcroppings, it was confirmed that human disturbances such as grazing activity had effects on the relative density and spatiotemporal distribution of the Brandt’s voles in MD [82].

### Sample collection

Traps were set before 6 a.m. and were collected after 8 a.m. in the morning. The details of each trapped vole were individually recorded (gender, body condition and body mass). Fecal samples were collected from each trap (no feces were gathered when traps contained more than one individual) during each trapping session. We sampled ear tissue with a biopsy ear punch and stored the tissue immediately in 95% ethanol until DNA isolation. Animals were handled and immediately released at the point of capture. The animal handling and sampling protocol followed the guidelines approved by the Institutional Animal Use and Care Committee, the Institute of Zoology, Chinese Academy of Sciences (CAS IAUCC).

### Parasite identification and counts

Fecal material collected from each individual was stored at 4°C overnight in Petri dishes on damp blotting paper to standardize the humidity content. Thereafter, each sample was weighed and the gastrointestinal parasite load measured; we measured the fecal egg counts (FEC; number of eggs per gram feces) using a McMaster floatation technique. Following the method of Schad, Ganzhorn and Sommer [61], we counted two chambers of a McMaster slide and used the mean values of the individual samples. We used potassium iodide in the flotation dilution, which enhances the detection of eggs with a higher specific weight, as proposed by Meyer-Lucht and Sommer [15]. We classified helminth eggs into morphotypes based on size and appearance and photographed them for taxonomic identification at a later stage.

### Microsatellite genotyping

For each vole, DNA extraction from the ear tissue sample was conducted using a TIANamp Genomic DNA Kit (Tiangen Biotech, Co., Ltd., Beijing, China) following the manufacturer’s protocol. All voles were typed for 10 microsatellites to assess neutral genetic diversity. These loci have previously been described for Brandt’s voles [83] and were amplified following the authors’ protocols [84].

### Screening of MHC variation

We examined variation in a highly polymorphic 200 bp fragment of exon 2 of the MHC class II DRB gene, which includes the functionally important antigen binding and recognition sites. As there was no sequence data available for Brandt's voles, primers were designed after alignment of the published sequences for small animals [17,48,85]; these were designated BVF (up): 5’-ATTACAACAACGGGACGCA-3’ and BVR (down): 5’-CTCGTAGTTGTGTCTGCA-3’. To identify suitable primers, an extensive blast search (http://www.ncbi.nlm.nih.gov) was carried out and DRB sequences from a wide range of animal species from different phylogenetic radiations were aligned. Amplifications were conducted in a final reaction volume of 50 μl, which included 15–50 ng of DNA, 0.25 mM of each primer, 200 mM of dNTPs, 5 μl of a 10 × reaction buffer solution and 0.5 U of Taq DNA polymerase (Beijing CoWin Bioscience Co., Ltd.). The PCR instrument used was a TaKaRa Thermal Cycler Dice TP600 (TaKaRa Bio Inc., Japan) and thermal cycling started with 3 min denaturation at 95°C, followed by 30 cycles at 95°C, 54°C and 72°C for 30 s each and ended with an elongation step at 72°C for 7 min.

Alleles were separated using SSCP [17]. SSCP is a sensitive method that can distinguish minimal allele differences; it has been widely used in human genetics and is popular in population genetics and evolutionary ecology [11,20,29]. PCR products were denatured at 98°C for 10 min and immediately transferred to ice for snap-cooling to produce single-strands and hinder reannealing. The ssDNA was then mixed with loading dye and loaded onto a non-denaturing 15% polyacrylamide gel according to the manufacturer’s protocol and with the following modifications: 12°C running temperature, pre-run for 5 min at 200 V maintained for 4 h at 100 V.

Gels were silver stained, scanned and processed with Quantity One (Bio-Rad Laboratories Inc., CA, USA) to align the individual band patterns. We counted bands with the same mobility as the same alleles and each of these alleles was sequenced at least once from both directions, and where possible twice, to confirm this assumption. Appropriate bands were cut from the polyacrylamide gel, eluted in Tris/borate/EDTA (TBE) buffer, and amplified using the same protocol described above. PCR products were gel purified and then sequenced.

### Estimates of genetic variation

To measure the overall neutral genetic diversity per population we used MLH [86], and mean microsatellite d^2^[87]. DRB sequences were revised manually using the BIOEDIT Sequence Alignment Editor [88] and aligned in GENEDOC version 2.6 [89]. We verified the sequence identity through homology with the published MHC alleles of other species using BLAST from NCBI with a cutoff E-value of 10^-6^. MEGA 5 [90] was employed to construct a phylogenetic tree of the DRB alleles based on the exon2 sequence, using the minimum evolutionary criteria [91], and to calculate the relative rates of nonsynonymous (d_N_) and synonymous (d_S_) substitutions according to the model of Nei and Gojobory [91] with the correction of Jukes and Cantor [92] for multiple hits. The d_N_/d_S_ rates were tested for significant differences with a Z-test. Calculations were carried out separately for ABS and non-ABS, assuming concordance with antigen binding sites in the human HLA class II molecule, DR1 [19].

MHC genetic diversity was described by the observed heterozygosity and the allelic richness. As the observed number of alleles in a sample is highly dependent on the number of individuals sampled, we calculated the allelic richness corrected in the different sample sizes using a rarefaction index implemented in FSTAT [93]: thereby, the expected number of alleles in each sub-sample is calculated for the number of individuals present in the smallest sample.

For both markers, differentiation across all populations and between population pairs was tested using GenePop 4.0 [94] for microsatellites and Arlequin 3.0 [95] for MHC. In addition, both global and pairwise estimate of F_ST_ were estimated using Arlequin following Weir and Cockerham [96]. Observed and expected heterozygosity for both markers were calculated by Arlequin. Linkage disequilibrium between pairs of loci and deviations from Hardy–Weinberg equilibrium for each locus were also tested in Arlequin.

### Statistical treatment

We used three measurements to describe the parasite burden: 1) parasite prevalence (for individuals, presence/absence of a given species; for populations, the percentage of the animals infected); 2) species richness, which was defined as the number of all of the parasite species present in one host; and 3) infection intensity, which was estimated using nematode fecal egg counts (FEC = log_10_ EPG; EPG: eggs/g feces).

Dissimilarities in parasite community composition between all host populations were assessed by calculating Hellinger distances using the R library vegan [97]. Hellinger distances are based on square-rooted proportional abundances [98] and, therefore, reflect relative differences in parasite community composition. A permutational multivariate analysis of variance on Hellinger distances was performed using the Adonis function in vegan [99]. Statistical significance was obtained through 1000 permutations of the data. This analysis was then repeated for each pair of host populations among MD or DWQ separately, applying a significance level corrected for multiple comparisons (3 pairwise comparisons, α = 0.017).

To investigate associations between multiple nematode infestation and the gene constitutions considering both type of markers on the population level, we used GLMMs. Models were fitted for overall mean species richness and overall mean parasite infection intensity. The models for species richness were calculated using a Poisson distribution and log-linear-link function. In the mean infection intensity models we applied a Gaussian error distribution with an identity link function. Owing to the small number of populations (n = 6), and to avoid colinearity, the four predictors of genetic diversity (microsatellite MLH, microsatellite d^2^, MHC heterozygosity, MHC alleles richness) were included in separate, but otherwise identical, models.

In order to test for possible associations and interactions between MHC gene and parasite loads, as well as finding support for parasite-driven selection mechanisms at the individual level, we also used GLMMs following the methods of Froeschke and Sommer [28]. We took (a) the prevalence (presence/absence) and (b) the infection intensity (FEC) data as response variables. For the prevalence data, logistic regression models were applied with a binomial error distribution and logit link function. For the log-transformed infection intensity data, we used a Gaussian error distribution with an identity link function. To analyze relationships between genetic constitution (heterozygote advantage) and intestinal nematode susceptibility, we included the MHC genotype (homozygote or heterozygote) and microsatellite MLH for each individual as fixed factors in our model. Heterozygote host individuals and animals with a higher allele divergence should be able to recognize a broader spectrum of parasites and thus potential lower prevalence and FEC rates would be interpreted as an advantage. To test the impact of specific MHC alleles (rare allele advantage and fluctuating selection) on nematode burden, we continued with the three most abundant nematode (*S*. *obvelata*, *A*. *tetraptera* and *Trichostrongylidae* family). As predictors we included the presence/absence of specific MHC alleles observed in more than five individuals as fixed factors. Simplification carried out by removing variables in the order of non-significance derived the model: Parasite load (prevalence or infection intensity) ~ specific MHC allele (present or absent) + Sex + Body mass. Negative associations may be interpreted as indicating alleles conferring resistance to the parasite species, whereas positive associations indicate susceptibility to the parasite species. To examine spatial variation in individual pathogen load and specific MHC alleles, the above analysis was then repeated for each region (MD or DWQ) separately. Because parasite load is probably influenced by individual sex and body mass, we included them as explanatory variables in all of the above GLMM analyses. We included ‘population’ as a random factor in our models to consider extra sources of variation in variances through the influences of different populations and, accordingly, geographical position of each individual.

Statistical analyses were performed using the R 2.14 statistical package [100]. We applied a modified false discovery rate procedure [101] to estimate the critical p value for the effect of MHC alleles. This procedure is an alternative to the Bonferroni correction and regarded as the best practical solution to the problem of multiple comparisons [102].

## Abbreviations

ABS: Antigen-binding site; dS: Number of synonymous substitutions per synonymous site; dN: Number of nonsynonymous substitutions per non synonymous site; DWQ: East ujimqin; EPG: Eggs/g feces; FEC: Fecal egg counts (log_10_ EPG); GLMMs: General linear mixed models; MD: Maodeng livestock farm; MHC: Major histocompatibility complex; MLH: Multilocus heterozygosity; SSCP: Single-stranded conformation polymorphism.

## Competing interests

Both authors declare that they have no competing interests.

## Authors’ contributions

HHX supervised the study. HHX and ZM designed the field and parasitological work. ZM collected samples, performed molecular techniques, phylogenetic analyses and analyzed output data. ZM wrote the first draft of the manuscript, and both authors contributed substantially to revisions and approved the final manuscript.

## Supplementary Material

Additional file 1: Table S1 MHC class II *DRB* alleles identified by the corresponding nucleotide sequences and the corresponding GenBank accession numbers. Click here for file
