# Supplementary material for: Parasite-mediated selection of major histocompatibility complex variability in wild brandt’s voles (Lasiopodomys brandtii) from Inner Mongolia, China
Source: BMC Evol Biol. 2013 Jul 12;13:149. doi: 10.1186/1471-2148-13-149 (PMC3720540; doi:10.1186/1471-2148-13-149)
Supplement: Additional file 1: Table S1 — MHC class II DRB alleles identified by the corresponding nucleotide sequences and the corresponding GenBank accession numbers. [file 1471-2148-13-149-S1.doc]

# Additional files

**Additional file 1:**

**Table S1-**MHC class II *DRB* alleles identified by the corresponding nucleotide sequences and the corresponding GenBank accession numbers.

| **Nucleotide sequence**  **MHC-*DRB* allele** | **GenBank**  **accession numbers** |
| --- | --- |
| *Labr*-DRB*01 | [JX046707](http://www.ncbi.nlm.nih.gov/nuccore/JX046707) |
| *Labr*-DRB*02 | [JX046708](http://www.ncbi.nlm.nih.gov/nuccore/JX046708) |
| *Labr*-DRB*03 | [JX046709](http://www.ncbi.nlm.nih.gov/nuccore/JX046709) |
| *Labr*-DRB*04 | [JX046710](http://www.ncbi.nlm.nih.gov/nuccore/JX046710) |
| *Labr*-DRB*05 | [JX046711](http://www.ncbi.nlm.nih.gov/nuccore/JX046711) |
| *Labr*-DRB*06 | [JX046712](http://www.ncbi.nlm.nih.gov/nuccore/JX046712) |
| *Labr*-DRB*07 | [JX046713](http://www.ncbi.nlm.nih.gov/nuccore/JX046713) |
| *Labr*-DRB*08 | [JX046714](http://www.ncbi.nlm.nih.gov/nuccore/JX046714) |
| *Labr*-DRB*09 | [JX046715](http://www.ncbi.nlm.nih.gov/nuccore/JX046715) |
| *Labr*-DRB*10 | [JX046716](http://www.ncbi.nlm.nih.gov/nuccore/JX046716) |
| *Labr*-DRB*11 | [JX046717](http://www.ncbi.nlm.nih.gov/nuccore/JX046717) |
| *Labr*-DRB*12 | [JX046718](http://www.ncbi.nlm.nih.gov/nuccore/JX046718) |
| *Labr*-DRB*13 | [JX046719](http://www.ncbi.nlm.nih.gov/nuccore/JX046719) |
| *Labr*-DRB*14 | [JX046720](http://www.ncbi.nlm.nih.gov/nuccore/JX046720) |
| *Labr*-DRB*15 | [JX046721](http://www.ncbi.nlm.nih.gov/nuccore/JX046721) |
| *Labr*-DRB*16 | [JX046722](http://www.ncbi.nlm.nih.gov/nuccore/JX046722) |
| *Labr*-DRB*17 | [JX046723](http://www.ncbi.nlm.nih.gov/nuccore/JX046723) |
| *Labr*-DRB*18 | [JX046724](http://www.ncbi.nlm.nih.gov/nuccore/JX046724) |
| *Labr*-DRB*19 | [JX046725](http://www.ncbi.nlm.nih.gov/nuccore/JX046725) |
